# Uncontrolled maternal chronic respiratory diseases in pregnancy: A new potential risk factor suggested to be associated with anorectal malformations in offspring

**DOI:** 10.1002/bdr2.1429

**Published:** 2018-12-19

**Authors:** Romy van de Putte, Ivo de Blaauw, Rianne Boenink, Monique H.E. Reijers, Paul M.A. Broens, Cornelius E.J. Sloots, Arno F.J. van Heijst, Marleen M.H.J. van Gelder, Nel Roeleveld, Iris A.L.M. van Rooij

**Affiliations:** ^1^ Department for Health Evidence Radboud Institute for Health Sciences, Radboud university medical center (Radboudumc) Nijmegen The Netherlands; ^2^ Department of Surgery—Paediatric surgery Radboudumc Amalia Children's Hospital Nijmegen The Netherlands; ^3^ Department Dekkerswald Radboudumc The Netherlands; ^4^ Department of Surgery, Division of Pediatric Surgery University of Groningen, University Medical Center Groningen Groningen The Netherlands; ^5^ Department of Pediatric Surgery Sophia Children's Hospital, Erasmus Medical Center Rotterdam The Netherlands; ^6^ Department of Pediatrics—Neonatology Radboudumc Amalia Children's Hospital Nijmegen The Netherlands; ^7^ REshape Innovation Center Radboudumc Nijmegen The Netherlands

**Keywords:** anal atresia, anti‐asthmatic drugs, congenital malformations, pregnancy, uncontrolled asthma

## Abstract

**Background:**

Chronic respiratory diseases and use of antiasthmatic medication during pregnancy may both play a role in the etiology of congenital anorectal malformations (ARM). However, it is unclear, whether the medication use or the underlying condition would be responsible. Therefore, our aim was to unravel the role of maternal chronic respiratory diseases from that of antiasthmatic medication in the etiology of ARM.

**Methods:**

We obtained 412 ARM patients and 2,137 population‐based controls from the Dutch AGORA data‐ and biobank. We used maternal questionnaires and follow‐up telephone interviews to obtain information on chronic respiratory diseases, antiasthmatic medication use, and potential confounders. Multivariable logistic regression analyses were performed to estimate odds ratios (ORs) with 95% confidence intervals (95% CI).

**RESULTS:**

We observed higher risk estimates among women with chronic respiratory diseases with and without medication use (1.4 [0.8–2.7] and 2.0 [0.8–5.0]), both in comparison to women without a chronic respiratory disease and without medication use. Furthermore, increased ORs of ARM were found for women using rescue medication (2.4 [0.8–7.3]) or a combination of maintenance and rescue medication (2.5 [0.9–6.7]). In addition, increased risk estimates were observed for women having nonallergic triggers (2.5 [1.0–6.3]) or experiencing exacerbations during the periconceptional period (3.5 [1.4–8.6]).

**CONCLUSIONS:**

Although the 95% CIs of most associations include the null value, the risk estimates all point towards an association between uncontrolled chronic respiratory disease, instead of antiasthmatic medication use, with ARM in offspring. Further in‐depth studies towards mechanisms of this newly identified risk factor are warranted.

## 1 INTRODUCTION

Anorectal malformations (ARM) are one of the most frequently observed birth defects of the digestive system and are characterized by a disturbed embryonic hindgut development during weeks 4–8 after conception. The prevalence ranges from 2 to 7 patients per 10,000 pregnancies worldwide, (InternationalClearinghouse, 2014) with severity ranging from ARM with or without perineal fistula to complex cloacal malformations (Holschneider et al., 2005). Additional birth defects, such as defects of the genitourinary system or defects that are part of the VACTERL (Vertebral, Anal, Cardiac, Tracheo‐Esophageal, Renal, and Limb defects) association, are observed in approximately 50% of ARM patients (Cuschieri & EurocatWorkingGroup, 2002; Stoll, Alembik, Dott, & Roth, 2007). Several nongenetic factors have been associated with ARM, such as assisted reproductive techniques (ART), twin pregnancies, maternal overweight, preexisting diabetes, and previous miscarriage (van de Putte et al., 2017; Wijers, van Rooij, et al., 2014). However, as these factors do not explain ARM in the majority of patients (Wijers, van Rooij, et al., 2014), the knowledge about the etiology of ARM is still far from complete.

Asthma is a common chronic disease among pregnant women (Kwon, Belanger, & Bracken, 2003; Murphy, 2015). Maternal asthma seems to be associated with increased risks of adverse birth outcomes, such as preterm birth, low birthweight, and birth defects, specifically of the central nervous system, respiratory system, digestive system, as well as cleft lip with or without cleft palate (Blais & Forget, 2008; Blais, Kettani, Elftouh, & Forget, 2010; Demissie, Breckenridge, & Rhoads, 1998; Murphy et al., 2013). As asthma exacerbations occur in approximately 45% of women with asthma during pregnancy, guidelines emphasize asthma management to prevent exacerbations (Murphy, 2015; NAEPP expert panel report, 2005; National Asthma Education and Prevention Program Asthma and Pregnancy Working Group, 2005). However, the influence of asthma and antiasthmatic medication on the risks of ARM in offspring remains unclear. Wijers et al. (2013) showed an increased risk of ARM in offspring among women with chronic obstructive lower pulmonary diseases, for example, asthma and bronchitis, in a registry‐based study. Zwink, Rissmann, Potzsch, Reutter, and Jenetzky (2016) showed a stronger association between ARM in offspring and maternal respiratory diseases. However, the results of both studies may have been influenced by antiasthmatic medication use among women with respiratory diseases.

The use of antiasthmatic medication during pregnancy is generally considered safe (Blais, Beauchesne, Rey, Malo, & Forget, 2007; Eltonsy, Kettani, & Blais, 2014). Nevertheless, several studies showed an increased risk of ARM (Garne et al., 2016; Kallen & Otterblad Olausson, 2007; Lin et al., 2012). Kallen & Otterblad Olausson (2007) showed an association between ARM and the use of antiasthmatic medication in early pregnancy, especially inhaled corticosteroid use, in a Swedish cohort study (1995–2004). In a subsequent study in the same population (1996–2011), however, the risk estimate was reduced (Kallen, 2014). Lin et al. (2012) detected an increased risk of isolated ARM in women using antiasthmatic medication in the periconceptional period, as did Garne et al. (2016) in a meta‐analysis of three cohort studies.

These studies claimed that it was difficult to disentangle the role of chronic respiratory diseases in these associations from that of their pharmacological treatment. Therefore, we performed a case–control study to investigate the association between maternal chronic respiratory diseases and ARM in offspring separately from the association between antiasthmatic medication and the occurrence of ARM.

## 2 METHODS

### 2.1 Study population

We obtained patients with ARM and healthy controls from the AGORA (Aetiologic research into Genetic and Occupational/environmental Risk factors for Anomalies in Children) data‐ and biobank of the Radboud university medical center (Radboudumc) in the Netherlands. AGORA includes clinical and questionnaire data, as well as DNA samples from children with birth defects or childhood cancer, population‐based control children, and their parents (van Rooij et al., 2016). A pediatric surgeon, a clinical geneticist, and the researchers collaboratively reviewed the medical records of ARM patients to obtain clinical information on ARM, the diagnosis of other birth defects, and known genetic causes. The ARM phenotypes were classified according to the Krickenbeck criteria (Holschneider et al., 2005). The EUROCAT classification for birth defects was used to differentiate between additional minor and major birth defects that were present within our patients (EUROCAT Guide 1.3 and reference documents, 2005).

From 2007 onward, pediatric surgeons asked parents of ARM patients to participate in AGORA at the child's first visit to the Radboudumc. They were invited by regular mail when their child was treated in the Pediatric Surgery Department of the Erasmus Medical Center Rotterdam or the University Medical Center Groningen. The parents were asked to fill out a questionnaire concerning information on demographics, family history, and health and lifestyle before and during pregnancy. We excluded ARM patients with a family history of ARM, a known genetic syndrome, a chromosomal abnormality, or a cloacal extrophy, whereas we included ARM patients with other birth defects in the study. Our population‐based controls were randomly sampled in 2010–2011 via 39 municipalities in geographical areas comparable to those of the patients. They were born between January 1990 and March 2011 and were of similar age as our patient group. Based on the questionnaire data, we excluded controls with major birth defects, a known genetic syndrome, or a chromosomal abnormality.

The Regional Committee on Research Involving Human Subjects Arnhem‐Nijmegen approved the AGORA study protocol in 2006, and all participants and/or their parents provided written informed consent.

### 2.2 Data collection

Maternal questionnaires provided information about the presence of chronic respiratory diseases, such as asthma or chronic bronchitis, and the use of antiasthmatic medication. We asked the women about the presence of any chronic disease in the 3 months before or during pregnancy. In addition, they were asked whether medication for asthma and/or chronic bronchitis was used, including the name of the medication, and the specific period in pregnancy in which the medication was used.

In addition, we conducted telephone interviews with women who reported a chronic respiratory disease and/or antiasthmatic medication use, and with women for whom this information remained unclear in the questionnaire. We assumed that women who clearly reported no chronic respiratory disease and no use of antiasthmatic medication did not have a chronic respiratory disease, so these women were not interviewed. The interview focused on clarifying missing information and obtaining more detailed information not yet assessed in the questionnaire, such as the self‐reported frequency of periconceptional antiasthmatic medication use (rescue use vs. maintenance use), type of trigger (allergic vs. nonallergic), and exacerbations in the periconceptional period (defined as no control over the chronic respiratory disease in the 3 months before pregnancy, deterioration of the respiratory disease during the first trimester, or experiencing asthma complaints during the entire periconceptional period). Within this study, the periconceptional period was defined as the 3 months before conception until the 10th week of pregnancy, as anorectal development is complete by that time (Schoenwolf et al., 2015). The antiasthmatic medication was reported to be used to treat asthma or chronic bronchitis and included inhaled corticosteroids, beta2‐adrenergic agonists, anticholinergics, cromoglicic acids, and leukotriene receptor antagonists. Antiallergic medication was not included as antiasthmatic medication.

As both the maternal questionnaire and the telephone interview generated self‐reported data, we combined these two data sources to assess the presence of chronic respiratory diseases and antiasthmatic medication use. In case of discrepancies, we assumed the telephone interview to be more reliable, because the latter was based on more in‐depth questions.

We extracted information on infant and maternal characteristics from the maternal questionnaires. Infant characteristics included gender, year of birth, descent (Dutch vs. non‐Dutch), low birthweight (<2,500 g), and preterm delivery (<37 weeks). Maternal characteristics were age at delivery, maternal education (low: no, primary, lower vocational, or intermediate secondary education; middle: intermediate vocational or higher secondary education; high: higher vocational or academic education), twin pregnancy (vs. singleton pregnancy), parity (primiparity vs. multiparity), previous miscarriage, ART, recommended folic acid use (use of folic acid supplements or multivitamins containing folic acid from 4 weeks before until 10 weeks after conception), periconceptional smoking (any smoking), preexisting diabetes mellitus, and pre‐pregnancy body mass index (BMI). BMI was calculated using the reported weight (in kilograms) and height (in meters) before conception and was categorized in four groups (<18.5: underweight; 18.5–24.9: healthy weight; 25.0–29.9: overweight; and ≥30: obese).

### 2.3 Statistical analyses

We used univariable and multivariable logistic regression analyses to estimate crude and adjusted odds ratios (ORs) with 95% confidence intervals (95% CIs) for independent associations between ARM in offspring and maternal chronic respiratory diseases and antiasthmatic medication use. To unravel the roles of chronic respiratory diseases and antiasthmatic medication use, we subdivided the women into four categories: (a) having a chronic respiratory disease without antiasthmatic medication use, (b) antiasthmatic medication use without having a chronic respiratory disease, (c) having a chronic respiratory disease with antiasthmatic medication use, and (d) having no chronic respiratory disease nor antiasthmatic medication use (reference group). Antiasthmatic medication use was subdivided into maintenance use, rescue use, and a combination of maintenance and rescue use. The type of medication was not taken into account, only the self‐reported frequency of use. We also studied the associations of ARM with exacerbations in the periconceptional period and with the type of trigger. We considered year of birth, maternal age at delivery, maternal education, primiparity, previous miscarriage, ART, recommended use of folic acid supplements, periconceptional smoking, and pre‐pregnancy BMI as potential confounders in the analyses, as these factors were previously identified as risk factors for ARM or were found to be associated with ARM in this study.

Potential confounders that changed the crude OR in bivariable analyses were included in the multivariable models, from which they were excluded when the OR did not change more than 10% from the fully adjusted OR upon removal. Only pre‐pregnancy BMI was found to be a true confounder in the analyses. Statistical analyses were performed using SPSS 22.0 for Windows (IBM SPSS, Chicago, IL).

## 3 RESULTS

In total, we included 412 ARM patients and 2,137 population‐based controls with available maternal questionnaire data. The majority of ARM patients had a perineal fistula (54%), followed by rectourethral fistulas (14%), vestibular fistulas (13%), ARM without fistula (4%), anal stenosis (3%), cloaca (2%), and rare/other types of ARM (9%). In total, 178 patients (43%) had additional major birth defects, mainly those that are also part of the VACTERL association.

No substantial differences were observed between patients and controls regarding gender, descent, twin pregnancies, parity, recommended folic acid supplement use, and age of the child when completing the questionnaire by the parents (Table 1). However, both the patients and their mothers were slightly younger at time of study, and at delivery, patients more often had a low birthweight or were delivered preterm, and patient mothers were lower educated and more often overweight or obese. In addition, previous miscarriage, fertility treatment, periconceptional smoking, and preexisting diabetes mellitus were more frequently observed in patient mothers, although the differences were generally small.

**Table 1:** Characteristics of patients with anorectal malformations, healthy controls, and their mothers

	Patients (*N* = 412), *N* (%)^a^	Controls (*N* = 2,137), *N* (%)^a^
*Infant characteristics*		
Boy	201 (48.8)	1,047 (49.0)
Year of birth		
≤1990	12 (2.9)	67 (3.1)
1991–1995	79 (19.2)	450 (21.1)
1996–2000	99 (24.0)	538 (25.2)
2001–2005	90 (21.8)	502 (23.5)
2006–2010	110 (26.7)	576 (27.0)
≥2011	22 (5.3)	4 (0.2)
Non‐Dutch descent	41 (10.3)	187 (8.8)
Low birthweight (<2,500 g)	64 (16.2)	150 (7.2)
Preterm delivery (<37 weeks)	71 (17.8)	185 (8.8)
*Maternal characteristics*		
Age at delivery		
<25 years	30 (7.3)	133 (6.3)
25–29 years	140 (34.0)	658 (30.9)
30–34 years	175 (42.5)	944 (44.4)
≥35 years	67 (16.3)	392 (18.4)
Educational level^b^		
Low	104 (25.3)	370 (17.4)
Middle	184 (44.8)	982 (46.1)
High	123 (29.9)	780 (36.6)
Twin pregnancy	18 (4.4)	79 (3.7)
Primiparity	193 (46.8)	955 (44.7)
Previous miscarriage	90 (22.1)	405 (19.0)
Assisted reproductive techniques	39 (9.5)	155 (7.3)
No folic acid supplement use^c^	143 (44.0)	713 (44.4)
Smoking^d^	107 (26.1)	498 (23.3)
Preexisting diabetes mellitus	10 (2.4)	12 (0.6)
Pre‐pregnancy body mass index		
Underweight (<18.5 kg/m^2^)	20 (5.1)	71 (3.5)
Normal (18.5–24.9 kg/m^2^)	243 (62.3)	1,436 (71.8)
Overweight (25.0–29.9 kg/m^2^)	87 (22.3)	364 (18.2)
Obese (≥30.0 kg/m^2^)	40 (10.3)	130 (6.5)
Age child at completion questionnaire in years, median (range)	8 (0.0–58.0)	10 (1.0–21.0)

a Numbers do not add up due to missing values (<1% for all variables, except for low birthweight [2.5%], preterm delivery [1.5%], folic acid use [24.2%, mainly due to missing values on the exact period of usage], and pre‐pregnancy body mass index [6.2%]).

b Low: no, primary, lower vocational, or intermediate secondary education; middle: intermediate vocational or higher secondary education; high: higher vocational or academic education.

c In the recommended period (4 weeks before until 10 weeks after conception).

d In the periconceptional period (3 months before until 10 weeks after conception).

Altogether, 102 women were eligible for the telephone interview (Supporting Information Figure 1). We were able to reach 77 (75%) of these women and observed 88% agreement between the questionnaire and telephone interview for the presence of chronic respiratory diseases and 96% agreement for the use of antiasthmatic medication. As mentioned before, we combined the information from the questionnaire with the telephone interview.

The risk of ARM seemed to be increased for offspring of women with chronic respiratory diseases (adjusted OR = 1.6 [0.9–2.6]) (Table 2). In addition, we observed a slightly elevated risk of ARM in offspring of women using antiasthmatic medication during the periconceptional period (adjusted OR = 1.4 [0.8–2.6]) (Table 2). When we only considered medication use during the first 2 months of pregnancy, in which the hindgut develops, similar results were obtained. More detailed analyses regarding the pharmaceutical components within the antiasthmatic medication was not possible, as the numbers became too low.

**Table 2:** Associations between anorectal malformations in offspring and maternal chronic respiratory diseases, antiasthmatic medication use during the periconceptional period, and the combination of both

	Patients, *N* (%)	Controls, *N* (%)	Crude OR^a^ (95% CI)	Adjusted OR^a^^,^ b (95% CI)
Presence of CRD				
No^c^	370 (94.9)	1,940 (97.0)	1.0	1.0
Yes	20 (5.1)	60 (3.0)	1.7 (1.0–2.9)	1.6 (0.9–2.6)
Use of antiasthmatic medication				
No^c^	376 (96.4)	1,950 (97.6)	1.0	1.0
Yes	14 (3.6)	47 (2.4)	1.5 (0.8–2.8)	1.4 (0.8–2.6)
Combination of CRD and medication use^d^				
Neither CRD nor medication use^c^	369 (94.6)	1,934 (96.8)	1.0	1.0
CRD/no medication use	7 (1.8)	16 (0.8)	2.3 (0.9–5.6)	2.0 (0.8–5.0)
No CRD/medication use	1 (0.3)	4 (0.2)	‐	‐
CRD/medication use	13 (3.3)	43 (2.2)	1.6 (0.8–3.0)	1.4 (0.8–2.7)

*Notes*. The periconceptional period ranges from 3 months before until 10 weeks after conception. CI: confidence interval; CRD: chronic respiratory disease; OR: odds ratio.

a We did not estimate ORs when <3 patients or controls were exposed.

b Adjusted for pre‐pregnancy body mass index.

c Reference category.

d Due to missing values, one patient and four controls could not be categorized in one of the groups.

After classification of women according to both their chronic condition and medication use, we observed higher risks among women having a chronic respiratory disease, with and without medication use (adjusted OR = 1.4 [0.8–2.7]) and adjusted OR = 2.0 [0.8–5.0]), both in comparison to the women without a chronic respiratory disease and without medication use (Table 2).The women with chronic respiratory diseases and medication use did not have an additional increased risk of ARM in offspring (OR = 0.8 [0.3–2.4]), compared to women with chronic respiratory diseases without medication use. The group of women in the category that used antiasthmatics without having a chronic respiratory disease (indications reported were milk allergy, incidental bronchitis, excessive mucus when waking up, and unknown in two cases) was too small to draw reliable conclusions on the risk of ARM in offspring.

Furthermore, we studied the chronic respiratory diseases and antiasthmatic medication use more in detail. Women using their medication on a rescue basis (mainly salbutamol, a short‐acting beta2 agonist), or women who reported a combination of maintenance and rescue use showed increased risks of ARM in offspring (adjusted OR = 2.4 [0.8–7.3] and adjusted OR = 2.5 [0.9–6.7]), compared to women without chronic respiratory diseases and no medication use (Table 3). The number of women using only maintenance medication was too small to estimate reliable ORs, but the percentage of women taking maintenance medication seemed to be comparable among patients and controls (0.5 vs. 0.7%).

**Table 3:** Associations between anorectal malformations in offspring and type of antiasthmatic medication used by the mother, type of trigger (allergic vs. nonallergic) for maternal chronic respiratory diseases, and presence of maternal exacerbations during the periconceptional period

	Patients, *N* (%)	Controls, *N* (%)	Crude OR^a^ (95% CI)	Adjusted OR^a^^,^ b (95% CI)
Type of antiasthmatic medication^c^				
Neither CRD nor medication^d^	373 (96.6)	1,945 (98.3)	1.0	1.0
Maintenance use	2 (0.5)	13 (0.7)	‐	‐
Rescue use	5 (1.3)	9 (0.5)	2.9 (1.0–8.7)	2.4 (0.8–7.3)
Maintenance and rescue use	6 (1.6)	12 (0.6)	2.6 (1.0–7.0)	2.5 (0.9–6.7)
Type of trigger^e^				
No CRD^d^	369 (95.8)	1,935 (97.8)	1.0	1.0
Allergic	4 (1.0)	20 (1.0)	1.0 (0.4–3.1)	1.0 (0.3–2.8)
Nonallergic	7 (1.8)	13 (0.7)	2.8 (1.1,7.1)	2.5 (1.0–6.3)
Allergic and non‐allergic	5 (1.3)	11 (0.6)	2.4 (0.8–6.9)	2.4 (0.8–7.0)
Presence of exacerbations^f^				
No CRD^d^	369 (96.1)	1,934 (97.9)	1.0	1.0
No	7 (1.8)	29 (1.5)	1.3 (0.6–2.9)	1.2 (0.5–2.8)
Yes	8 (2.1)	12 (0.6)	3.5 (1.4–8.6)	3.2 (1.3–8.0)

*Notes*. The periconceptional period ranges from 3 months before until 10 weeks after conception. CI: confidence interval; CRD: chronic respiratory disease; OR: odds ratio.

a We did not calculate ORs when <3 patients or controls were exposed.

b Adjusted for pre‐pregnancy body mass index.

c Due to missing values, five patients and 23 controls could not be categorized.

d Reference category.

e Due to missing values, six patients and 23 controls could not be categorized.

f Due to missing values, seven patients and 27 controls could not be categorized.

In addition, especially women with nonallergic triggers or a combination of allergic and nonallergic triggers seemed to have increased risks of having a child with ARM (adjusted OR = 2.5 [1.0–6.3] and adjusted OR = 2.4 [0.8–7.0]). In contrast, no association with ARM in offspring was observed for women with allergic triggers (adjusted OR = 1.0 [0.3–2.8]). Women who experienced exacerbations during the periconceptional period were three times more likely to have a child with ARM in comparison with the reference group (adjusted OR = 3.2 [1.3–8.0]), whereas no increased risk was observed among women with chronic respiratory diseases without exacerbations (adjusted OR = 1.2 [0.5–2.8]). To study the effect of exacerbations independently from rescue medication use, we additionally corrected for the frequency of medication use. This led to ORs for women with and without exacerbations in the same order of magnitude (OR = 2.7 [0.8–9.5] and OR = 1.0 [0.3–3.5]).

## 4 DISCUSSION

In this study, we tried to disentangle the role of chronic respiratory diseases and antiasthmatic medication use during the periconceptional period in the etiology of ARM. Increased risk estimates of ARM in offspring were observed for women with chronic respiratory diseases, especially women without periconceptional antiasthmatic medication use, in comparison with women who neither have a chronic respiratory disease nor use antiasthmatic medication. The risk estimate of ARM in offspring was not increased in women with chronic respiratory diseases who used antiasthmatic medication, compared to women with chronic respiratory diseases without medication use. Furthermore, increased risk estimates of ARM in offspring were observed in particular among women using their medication on a rescue basis, women having nonallergic triggers, and women having exacerbations during the periconceptional period. Although all but one of the 95% CIs contained the null value, these associations all suggest an association between ARM in offspring and an uncontrolled chronic respiratory disease state.

A strength of this study was the large number of representative and well‐characterized ARM patients and comparable population‐based controls. After applying several exclusion criteria, we observed additional birth defects in 43% of the patients, which corresponds with previous studies reporting 40–70% of ARM patients having additional birth defects (Cuschieri & EurocatWorkingGroup, 2002; Stoll et al., 2007; Wijers et al., 2013). Moreover, the distribution of ARM subtypes in our study was comparable with patients from the European ARM‐Net registry (de Blaauw et al., 2013). The population‐based controls were representative for the source population of the patients as they were randomly selected from the same geographical areas as the patients and were of similar age. There remains a possibility that the controls did not participate in a way that they remained representative after inclusion. However, most of their representative characteristics are similar to the general Dutch pregnant population (Wijers, de Blaauw, et al., 2014).

Another strength is that we conducted a telephone interview to obtain more detailed information about the chronic respiratory diseases and the periconceptional antiasthmatic medication use, enabling us to perform more in‐depth analyses. A limitation, however, is that we only interviewed women who reported chronic respiratory diseases or antiasthmatic medication use and women for whom this information was unclear in the questionnaire. As it was not feasible to also contact women who did not report positively on either of these variables, the presence of chronic respiratory diseases might have been underreported. However, we assume this is unlikely, as 98% agreement was observed for not reporting respiratory diseases in a study comparing obstetric records and questionnaires completed by pregnant women (van Gelder et al., 2015).

Another limitation was the possibility for recall bias, as the risk factors included in our study were self‐reported and especially because parents were asked to recall circumstances that occurred some time ago. The median age of the children at the time of filling out the questionnaire was a little bit lower in patients (8 years, range 0–58 years) than in controls (10 years, range 0–21 years; Table 1). Among the patients, there was only 1 patient with 58 years between childbirth and filling out the questionnaire; by excluding this outlier, the range is 0–22 years. Because of the small differences between patients and controls, and the fact that this variable was not a confounder in our analyses, we expect that differential misclassification due to recall times is minimal.

The prevalence of chronic respiratory diseases in the periconceptional period was only 3% among control mothers (vs. 5% in patient mothers). These numbers are lower than the general rate of these conditions in the Netherlands in 2016, approximately 6% (CBS, 2016), which is similar to the numbers reported in the United States (4–8%) (Kwon et al., 2003; Murphy, 2015). However, Kwon et al. demonstrated that the rate of asthma among pregnant women varies with time, population, and method of data collection (Kwon et al., 2003). According to the National Health Interview Survey for the time period 1997–2000, the total prevalence was 4%, compared to 8% in the time period 2000–2001 with the Behavior Risk Factor Surveillance System (Kwon et al., 2003). The relatively low prevalence of chronic respiratory diseases in our study may partly be explained by the fact that almost 50% of the women were pregnant before 2000. Unfortunately, we were not able to stratify by time to study whether timing is associated with the exposure, as the numbers will become too small to draw any reliable conclusions. In addition, it is possible that some women did have a chronic respiratory disease during childhood but did not experience any symptoms during pregnancy. The resulting underreporting is likely to be similar for our patient and control population, as maternal chronic respiratory diseases and associated exacerbations are not generally known as risk factors for birth defects, which may have resulted in an underestimation of the real effect.

Both antiasthmatic medication use and chronic respiratory diseases show increased risk estimates of ARM in offspring in the current study. Our risk estimates for the effects of antiasthmatic medication use on ARM in offspring (adjusted OR = 1.4 [0.8–2.6]) were comparable to those found in the studies by Kallen & Otterblad Olausson (2007) and Kallen (2014). Lin et al. (2012) detected a twofold increased risk of ARM in offspring, however, in isolated ARM and for anti‐inflammatory medication only. Garne et al. (2016) showed a threefold increased risk of ARM in offspring, but this study was conducted for inhaled corticosteroids only. For chronic respiratory diseases, Wijers et al. (2013) showed similar risks for chronic obstructive lower pulmonary diseases in a registry‐based study, compared to our risk estimate (adjusted OR = 1.6 [0.9–2.6]. Remarkably, Zwink et al. (2016) showed a 30‐fold increased risk of ARM in offspring for maternal chronic respiratory. The large difference with our study may be due to their inclusion of allergy to grass, pollen, or animal hair as respiratory diseases, whereas we did not include these allergies to limit heterogeneity. Based on the above‐mentioned results, we conclude that our study is in line with the existing literature. However, we went one step further by unraveling the effects of chronic respiratory diseases from those of medication use.

In our study, we observed elevated risk estimates for women who had chronic respiratory diseases and for women who used antiasthmatic medication, although the 95% CI did include the null value. In addition, women with a chronic respiratory disease without antiasthmatic medication use had the highest risk estimate of ARM in offspring. One could argue that these women stopped using their medication because they experienced fewer symptoms. However, the argument that we most often heard during the telephone interview was that women stopped using their medication because they were afraid to harm their unborn child. When we compared women with chronic respiratory diseases with and without medication use, no additional elevated risk was observed. Therefore, the results indicate that the chronic respiratory disease itself seems to be associated with ARM in offspring and not the associated medication use. However, were not able to draw any conclusions based on this information only.

The telephone interviews helped us to obtain more detailed information to support our findings of the chronic respiratory disease being responsible. Especially women who used rescue medication, had nonallergic triggers, and experienced exacerbations during the periconceptional period had increased risk estimates of ARM in offspring. To study the independent effect of exacerbations, we corrected for the frequency of medication use. However, we found results in the same order of magnitude, indicating that the increased ORs among women experiencing exacerbations can be attributed to the exacerbations itself and not to the use of rescue medication. Both, the presence of exacerbations and the use of rescue medication point toward uncontrolled chronic respiratory diseases. In addition, nonallergic triggers, such as weather conditions, viral infections, and outdoor and indoor pollutants, are generally harder to avoid than allergic triggers; therefore, women may also experience more problems with controlling their chronic respiratory disease. Although the CIs for rescue medication, a combination of rescue and maintenance medication, and nonallergic triggers do contain the null value, possibly due to small numbers, their lower limits were very close to 1, and they all point in the same direction. Therefore, we conclude that these results suggest that uncontrolled chronic respiratory diseases may be associated with ARM in offspring. Blais et al. showed that women with exacerbations are two times more likely to have a child with a major birth defect, after exclusion of women using oral corticosteroids (Blais & Forget, 2008). This study is in line with our finding that especially the underlying uncontrolled chronic respiratory disease seems responsible for the increased risks of ARM in offspring.

Proposed mechanisms for adverse pregnancy outcomes in women with an uncontrolled chronic respiratory disease are, for example, maternal hypoxia or the release of inflammation mediators (Blais et al., 2010; Murphy, Gibson, Smith, & Clifton, 2005). Previous studies showed that the number of interferon‐gamma‐producing cells and the levels of circulating heat shock protein‐70 were increased in asthmatic women compared to women experiencing an “uncomplicated” pregnancy, and that this increase was associated with fetal growth restriction (Tamasi et al., 2005; Tamasi et al., 2010). Maternal hypoxia might lead to adverse effects due to impaired fetal oxygenation (National Asthma Education and Prevention Program Asthma and Pregnancy Working Group, 2005). It is very important to mention that both of these hypothesized mechanisms might not only play a role in disturbing anorectal development but also the embryonic development of other organ systems. Therefore, we believe it is important to study the role of this newly identified risk factor in more detail.

In conclusion, even with the low frequency of women experiencing chronic respiratory diseases, our results suggest the involvement of uncontrolled maternal chronic respiratory diseases, with or without the use of rescue medication to alleviate exacerbations, in the development of ARM in offspring. This study suggested the involvement of uncontrolled maternal chronic diseases as a new mechanism in the etiology of birth defects that requires more in depth‐studies among larger groups of patients with all kinds of birth defects.

## CONFLICTS OF INTEREST

The authors declare no potential conflict of interests.
